# Towards Electrosynthesis in *Shewanella*: Energetics of Reversing the Mtr Pathway for Reductive Metabolism

**DOI:** 10.1371/journal.pone.0016649

**Published:** 2011-02-02

**Authors:** Daniel E. Ross, Jeffrey M. Flynn, Daniel B. Baron, Jeffrey A. Gralnick, Daniel R. Bond

**Affiliations:** 1 The BioTechnology Institute, University of Minnesota-Twin Cities, St. Paul, Minnesota, United States of America; 2 Department of Microbiology, University of Minnesota-Twin Cities, St. Paul, Minnesota, United States of America; New England Biolabs, Inc., United States of America

## Abstract

Bioelectrochemical systems rely on microorganisms to link complex oxidation/reduction reactions to electrodes. For example, in *Shewanella oneidensis* strain MR-1, an electron transfer conduit consisting of cytochromes and structural proteins, known as the Mtr respiratory pathway, catalyzes electron flow from cytoplasmic oxidative reactions to electrodes. Reversing this electron flow to drive microbial reductive metabolism offers a possible route for electrosynthesis of high value fuels and chemicals. We examined electron flow from electrodes into *Shewanella* to determine the feasibility of this process, the molecular components of reductive electron flow, and what driving forces were required. Addition of fumarate to a film of *S. oneidensis* adhering to a graphite electrode poised at −0.36 V *versus* standard hydrogen electrode (SHE) immediately led to electron uptake, while a mutant lacking the periplasmic fumarate reductase FccA was unable to utilize electrodes for fumarate reduction. Deletion of the gene encoding the outer membrane cytochrome-anchoring protein MtrB eliminated 88% of fumarate reduction. A mutant lacking the periplasmic cytochrome MtrA demonstrated more severe defects. Surprisingly, disruption of *menC*, which prevents menaquinone biosynthesis, eliminated 85% of electron flux. Deletion of the gene encoding the quinone-linked cytochrome CymA had a similar negative effect, which showed that electrons primarily flowed from outer membrane cytochromes into the quinone pool, and back to periplasmic FccA. Soluble redox mediators only partially restored electron transfer in mutants, suggesting that soluble shuttles could not replace periplasmic protein-protein interactions. This work demonstrates that the Mtr pathway can power reductive reactions, shows this conduit is functionally reversible, and provides new evidence for distinct CymA:MtrA and CymA:FccA respiratory units.

## Introduction

Reduction of external electron acceptors for dissimilatory respiration by Proteobacteria requires electron transfer proteins to link intracellular oxidative reactions to extracellular reductions beyond the outer membrane (OM). One γ-proteobacterium, *Shewanella oneidensis* strain MR-1, a facultative anaerobe, makes the connection to many external terminal electron acceptors [1], [2] through the Mtr respiratory pathway [3]. The complete Mtr pathway consists of a conduit [4], [5] of multiheme *c*-type cytochromes (MtrA and MtrC), a non-heme OM β-barrel (MtrB) that connects these cytochromes, and a cytoplasmic membrane (CM) associated quinol oxidase (CymA). Briefly, electrons from the menaquinone pool are passed to CymA, which is oxidized by the periplasmic face of the conduit (MtrA). Electrons subsequently traverse the OM and exit at MtrC [4], [5]. The OM-spanning MtrCAB complex has been extensively studied as the route for electron transfer out of the cell, and the terminal reductase MtrC is essential for reduction of soluble and insoluble metals [1], [6], [7], electron shuttles [8], and electrodes [8]–[12]. Furthermore, purified MtrC has been shown to have activity *in vitro* towards iron oxides [13]–[15], soluble electron shuttling compounds [14], and electrode surfaces [7], [16]. Recently, *E. coli* was engineered to reduce inorganic extracellular electron acceptors via expression of only *mtrC*, *mtrA*, and *mtrB* [17].

With the interest in “microbial electrosynthesis,” where biosynthetic pathways can be driven with electricity [18], mechanistic information of reverse electron transfer into bacteria is needed. The use of electrodes as electron donors has been examined previously in *Geobacter* spp. for partial reduction of nitrate [19], reduction of fumarate to succinate [19], reductive dechlorination of tetrachlorethene [20], reduction of U(VI) [21], and to drive acetogenic microorganisms in reduction of carbon dioxide to organic compounds [22]. However, in all of these cases, the complete pathway for electron uptake across multiple membranes to a terminal reductase was not determined.

To quantify inward electron flux, we have utilized electrode-attached *S. oneidensis* catalyzing the two-electron reduction of fumarate to succinate. *S. oneidensis* contains a soluble fumarate reductase localized to the periplasmic space, which is unique from the membrane-associated fumarate reductase in other bacteria (i.e. *Escherichia coli*) [23]. Under anaerobic conditions, FccA is the sole fumarate reductase in *Shewanella* [24], [25], and strongly favors the reductive reaction [26], [27]. In the absence of soluble electron shuttles, contact between electron transfer proteins and the electrode surface is essential for electron transfer in *S. oneidensis* [9]. This requirement, combined with sensitive electrochemical capabilities, was exploited to examine the hypothesis that the Mtr pathway could be reversed for cathodic electron uptake.

Using this electrochemical approach, combined with deletions of genes encoding cytochromes, structural proteins, and quinone biosynthesis, we determined that an intact OM protein complex (MtrCAB) is required for electrode-dependent fumarate reduction in *S. oneidensis*, and that the driving force required agrees with known potential-dependent responses of the fumarate reductase rather than the E′° of the fumarate/succinate couple (+0.03 V). Furthermore, our data revealed a surprising requirement for CymA and the menaquinone pool for inward electron flux to periplasmic acceptors, and suggests a mechanism involving distinct CymA respiratory units for fumarate reduction (CymA:FccA) and metal reduction (CymA:MtrA). Taken together, our findings show a potential ability to drive pathways with electricity (electrosynthesis) in *Shewanella* using the Mtr respiratory pathway and provides mechanistic information about electron transfer into the cell.

## Materials and Methods

### Reagents

Restriction enzymes, phosphatase, DNA polymerase mix, and T4 DNA Ligase were obtained from New England Biolabs (Ipswich, MA). TOPO TA cloning kit was obtained from Invitrogen (Carlsbad, CA). For PCR cleanup, gel extraction and plasmid preparation, QIAquick PCR Purification Kit, QIAquick Gel Extraction Kit and QIAprep Spin Miniprep Kit from Qiagen (Valencia, CA) were used respectively. Sodium fumarate, sodium lactate, and riboflavin were obtained from Sigma (St. Louis, MO).

### Bacterial strains, plasmids and growth conditions

*S. oneidensis* was previously isolated from Lake Oneida in New York [1]. Overnight cultures were inoculated using a single colony from freshly streaked plates in Luria-Bertani (LB) broth. Where noted, *Shewanella* Basal Medium (SBM) was composed as previously described [28]. Table 1 shows the strains and plasmids used in this study.

**Table 1:** Strains and plasmids used in this study.

Strain or Plasmid	Characteristics	Reference/Source
*S. oneidensis* strain MR-1	Isolated from L. Oneida, NY	[1]
*E. coli* strain UQ950	*E. coli* DH5α λ(*pir*) host for cloning	[47]
*E. coli* strain WM3064	Donor strain (DAP auxotroph) for conjugation	[47]
JG 686	*S. oneidensis* MR-1, Δ*fccA*	This study
JG 1064	*S. oneidensis* MR-1, Δ*cymA*	This study
JG 730	*S. oneidensis* MR-1, Δ*mtrA*	[8]
JG 700	*S. oneidensis* MR-1, Δ*mtrB*	[8]
JG 665	*S. oneidensis* MR-1, ΔPEC (*periplasmic electron carriers* Δ*mtrA*, Δ*mtrD*, Δ*cctA*, Δ*dmsE*, and Δ*SO4360)*	[32]
JG 300	*menC::mini-*Tn10 *nptII* Kan^r^	[27]
pSMV3	9.1-kb mobilizable suicide vector; *oriR6K, mobRP4, sacB*, Kan^r^ Ap^r^	[47]
pΔ*fccA*	2 kb deletion construct for *fccA* in pSMV3	This study
pΔ*cymA*	2 kb deletion construct for *cymA* in pSMV3	This study
**Primers**		
*fccA* UP Fwd*fccA* UP Rev*fccA* DN Fwd*fccA* DN Rev*cymA* UP Fwd*cymA* UP Rev*cymA* DN Fwd*cymA* DN Rev	NNACTAGTTGCAGCGGTGCTATTAA NNGAATTCCATTGCGCCAGAGATCA NNGAATTCATCGCGGGTGCATCTGC NNGAGCTCATGGCAGGCTGATAGGC CGGGATCCTGAGCGTTTCAGTGCCTT CGGAATTCAAATAGTGCACGCCAGTT CGGAATTCCCTATCCAAAAGGATAAG GGACTAGTCCGCATGTTGCCGTTGCA	This studyThis studyThis studyThis studyThis studyThis studyThis studyThis study

### Deletion constructs

*S. oneidensis* mutant strains were created as described previously [28]. Briefly, regions upstream and downstream of the gene-of-interest were ligated into pSMV3. Subsequent transformation into *E. coli* WM3064 mating strain, conjugation between the mating strain and *S. oneidensis* MR-1 and incubation under conditions selecting for removal of the target gene by recombination produced strains with deletions in the desired regions. The *menC* mutant, previously reported, was generated by transposon insertion using a suicide plasmid with a mini-Tn10 transposon derivative [29].

### Electrochemical techniques

Bioreactors (electrochemical cells) were prepared as described previously [30]. The bioreactor consisted of an AXF-5Q graphite (Poco Graphite Company, Decatur, TX) working electrode measuring 0.5 cm ×2 cm ×1 mm, a platinum wire cathode/counter, and a glass frit enclosed, saturated calomel reference electrode connected via a salt bridge (Fisher Scientific, Pittsburgh, PA), which was fitted into a Teflon top placed onto a 25 mL glass cone (Bioanalytical Systems, West Lafayette, IN). The working electrode was polished with 400-grit sandpaper, rinsed and cleaned in 1 N HCl for 16 hours. It was then attached to a platinum wire using a nylon screw and nut (Small Parts, Inc., Miramar, FL). The platinum wire was soldered to an insulated copper wire within a glass capillary tube. The reference electrode salt bridge was maintained with a 5 mm diameter glass capillary tube capped with a nanoporous vycor frit (Bioanalytical Systems, West Lafayette, IN) filled with 0.1 M sodium sulfate solution in 1% agarose connected to a larger tube which contained the reference electrode bathed in 0.1 M sodium sulfate. The bioreactors were monitored and potentials were maintained using a 16-channel VMP® potentiostat (Bio-Logic SA, Knoxville, TN). Anaerobic conditions were maintained with constant flushing of humidified nitrogen gas. The bioreactors were stirred and maintained at 30°C in a circulating water bath.

### Artificial biofilm formation and characterization

Thin films of attached cells were prepared as described previously with modifications [9]. Overnight cultures (10 mL of >1O.D. 600) were used to inoculate 400 mL of LB. LB cultures were shaken for 16 hours at 30°C. To facilitate anaerobic culture conditions, cultures were incubated for an additional 5 hours at 30°C without shaking. Cultures were then centrifuged at 7000× g for 10 minutes. Cell pellets were washed in 25 ml of SBM, centrifuged, and gently re-suspended in 10 mL of SBM. The resultant cell suspension was transferred to a sterile, anaerobic 3-electrode bioreactor containing a 2 cm^2^ graphitic working electrode. The working electrode was poised at an oxidizing potential of +0.24 V *versus* SHE for 16 hours to facilitate attachment of cells to electrodes. The bioreactors were then washed twice with sterile, anaerobic SBM and cyclic voltammetry (CV) was performed (sweeps from −0.56 to +0.44 V *versus* SHE) to determine baseline features for comparison to subsequent fumarate and flavin additions. The working electrode was poised at a reducing potential of −0.36 V *versus* SHE and current was monitored until a steady baseline was reached (∼1 hour). Fumarate was added to a final concentration of 50 mM and current was monitored.

### Determination of electrode-attached protein

To quantify attached biomass, electrodes were assayed for total protein as described previously [8], [9]. Briefly, electrodes were removed from the bioreactor, washed, and incubated in 1 mL of 0.2 N NaOH for 30 minutes at 90°C to solubilize attached protein. The supernatant was analyzed using the bicinchoninic acid (BCA) assay (Pierce, Rockford, IL) according to manufacturer's instructions.

## Results

### Electrode-dependent fumarate reduction by thin films of *S. oneidensis* requires FccA

**Figure 1:**
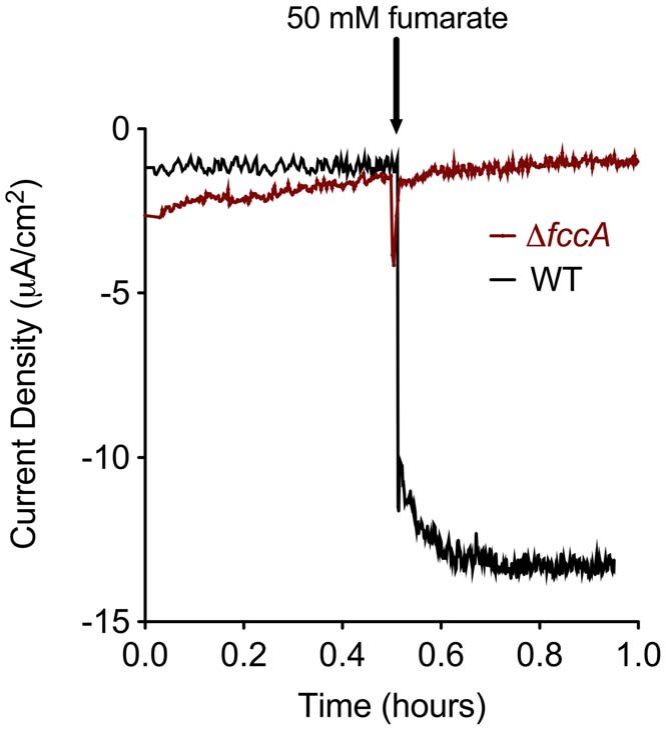
Electrode-dependent fumarate reduction in *S. oneidensis* MR-1. Representative chronoamperometry (CA) of *S. oneidensis* MR-1 thin films on graphite electrodes. Electrodes were poised at −0.36 V *versus* SHE and after 0.5 hours 50 mM fumarate was added to stirred bioreactors.

Our goal was to identify components necessary and sufficient for inward electron flux from an electrode surface into *S. oneidensis*. Using established electrochemical techniques [9] thin films of *S. oneidensis* attached to a graphite electrode in the absence of added soluble shuttles were analyzed for their ability to catalyze the reduction of fumarate to succinate. When electrodes were poised at a reducing potential (−0.36 V *versus* SHE), continuous amperometry measurements showed a sudden onset of cathodic current upon addition of 50 mM fumarate to stirred anaerobic bioreactors, where wild type *S. oneidensis* reached an average net current density of −17.2+/−4.4 µA/cm^2^ (n = 6, Fig. 1). The observed negative current was indicative of electron flow from the electrode into attached cells. Furthermore, abiotic controls showed no electrochemical response upon fumarate addition (data not shown). These experiments verified electron uptake capabilities of *S. oneidensis* attached to an electrode, and demonstrated the repeatability of the artificial biofilm technique.

**Figure 2:**
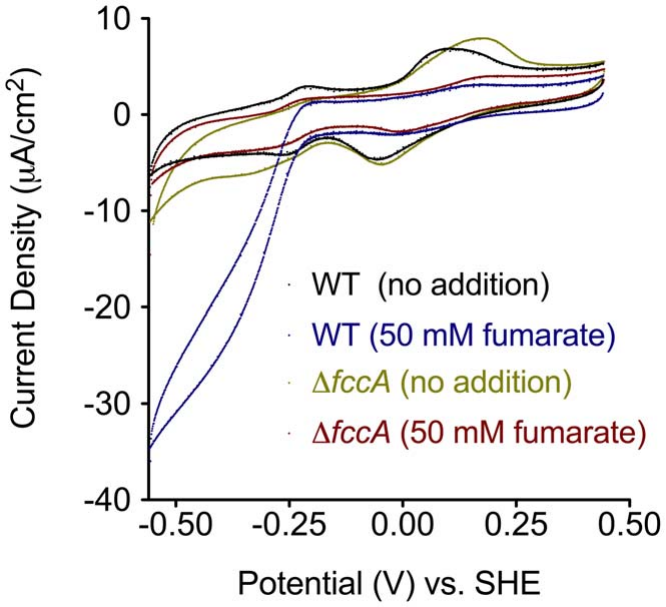
Single turnover and catalytic voltammetry of WT and Δ*fccA* thin films attached to electrodes. Representative cyclic voltammograms (1 mV/s) of fumarate responses of *S. oneidensis* MR-1 (black trace, no addition; blue trace, 50 mM fumarate) and Δ*fccA* (olive trace, no addition; red trace, 50 mM fumarate) after 16 hr of attachment to electrodes poised at +0.24 V *versus* SHE. The redox peak centered at +0.2 V *versus* SHE is indicative of a redox active species in close proximity to the electrode surface, i.e. *c*-type cytochromes exposed on the outer membrane.

We next tested whether the observed electrochemical response was linked to the periplasmic fumarate reductase. In mutants lacking *fccA*, no sustained increase in current density was observed upon fumarate addition (Fig. 1), which further supported the conclusion that electrochemical responses were a direct measure of electron flow from the electrode into the periplasm for reduction of fumarate. To characterize electrode-linked fumarate reduction across a range of imposed potentials, slow scan rate cyclic voltammetry (CV) was performed in the presence and absence of fumarate (Fig. 2). Voltammograms of wild type or Δ*fccA* thin films in the absence of fumarate (single turnover) showed no significant differences, most notably in the potential range of outer membrane cytochromes [9], [11]. However, upon addition of fumarate a catalytic waveform with a midpoint potential centered at −0.26 V *versus* SHE, and a secondary boost at lower potentials (below −0.3 V) was observed in wild type cells. The *fccA* mutant showed no response, even at lower potentials (Fig. 2).

### Reversible electron transfer is dependent upon outer membrane and periplasmic cytochromes

**Figure 3:**
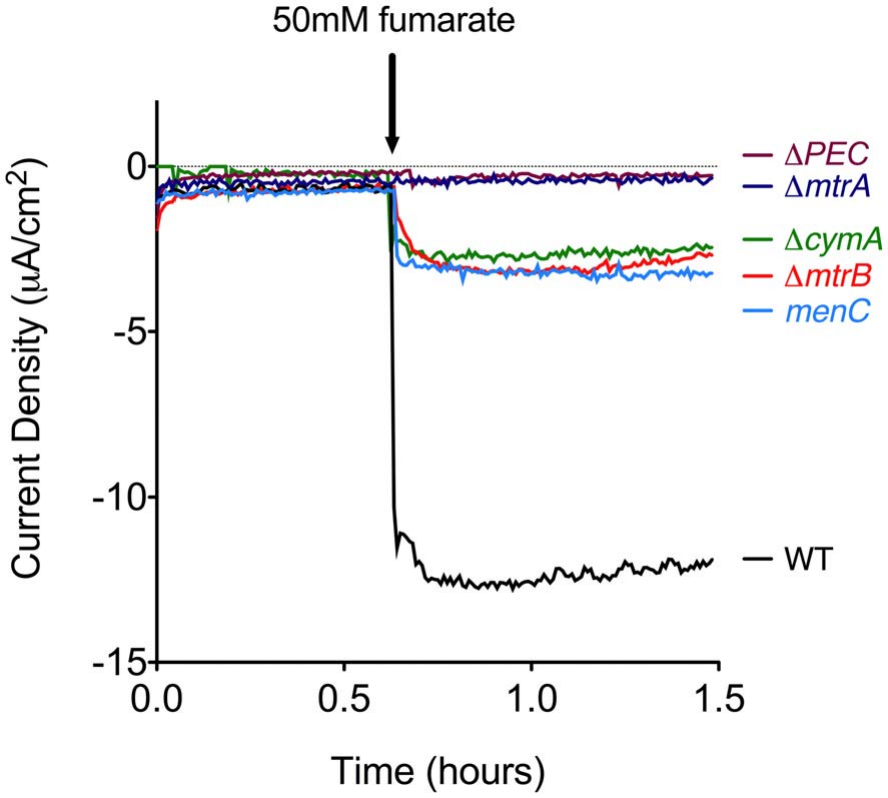
Components of the Mtr pathway are required for inward electron flux. Representative chronoamperometry of Mtr mutant thin films on electrodes poised at −0.36 V *versus* SHE. Current was constantly monitored and at 0.6 hours, 50 mM fumarate was added to stirred bioreactors.

We next sought to investigate the role of the Mtr pathway in reversible electron transfer. Various mutants previously characterized for reduction of Fe(III) or soluble electron shuttles [8], [10], were tested for their electrode-dependent fumarate reduction capabilities (Fig. 3). Since the Mtr pathway spans the cytoplasmic and outer membranes, we individually examined the importance of periplasmic and OM components.

**Figure 4:**
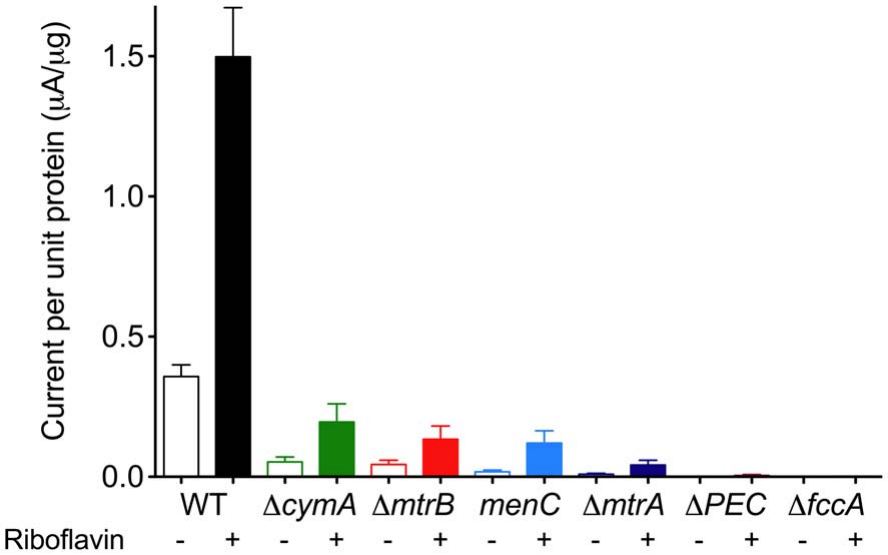
Rate of inward electron flux normalized to total electrode-attached protein, showing effect of riboflavin. Maximum current responses after fumarate addition (open bars) were normalized to attached protein values to obtain a specific rate of electron transfer (µA/µg protein; n≥3, +/− standard deviation). Closed bars represent maximum current values after addition of 1 µM riboflavin.

Beginning with the outer surface, we exploited the fact that MtrB, a putative integral outer membrane β-barrel, is required for proper localization of MtrC and OmcA to the OM [31]. Deletion of the *mtrB* gene eliminated most of the fumarate-dependent electron uptake current (Fig. 3). For a more quantitative assessment of electron transfer rates, all current values were normalized to total electrode-attached protein (µA/µg protein), to correct for differences in cell attachment between mutants. The *mtrB* mutant had a specific current that was 12% of the wild type rate (+/−1.7%, n = 3, Fig. 4). These findings were consistent with previous studies showing MtrB was required for outward electron transfer to electrodes [8], [10].

To test the role of periplasmic components, soluble periplasmic cytochromes were deleted. In particular, two mutant strains were examined: Δ*mtrA*, and a mutant devoid of all known periplasmic electron carriers (*mtrA, mtrD, cctA, dmsE*, and *SO4360*) termed Δ*PEC* [32]. Deletion of *mtrA* resulted in an almost complete loss of specific activity (3% of wild type, +/−1% n = 3 Fig. 4) consistent with the requirement of MtrA in Fe(III), Mn(IV) oxide [4], [6], [10] and electrode reduction [8], [10]. Likewise, Δ*PEC* showed even less electrode-dependent fumarate reduction (0.4% of wild type, +/−0.1% n = 3 Fig. 4). These mutants highlighted the need for a periplasmic electron carrier to complete the electrochemical circuit to FccA.

### The majority of electron flux to FccA proceeds via CymA and the menaquinone pool

Recent work with purified proteins has shown evidence for a direct interaction between the periplasmic cytochrome MtrA and FccA [33]. If this interaction were sufficient to direct electrons from the outer surface to FccA, CM localized proteins would not be needed for inward electron flux. However, mutants lacking *cymA* were severely impaired in their ability to reduce fumarate. With a specific current of 15% of wild type (+/−3%, n = 3), Δ*cymA* was not completely inactive, suggesting residual electron transfer between MtrA and FccA or MtrA and an unknown CM or periplasmic protein *in vivo*. Heme staining of whole cell extracts revealed no differences in cytochrome expression compared to wild type (data not shown) confirming that the inability of a *cymA* mutant to reduce fumarate was due to the absence of CymA and not differential expression of the Mtr pathway. CymA has only been shown to be required for electron flow from dehydrogenases in the CM to periplasmic enzymes (such as FccA) [34]–[36] yet our data showed that under these conditions, the route of electron flow from the outer surface to periplasmic fumarate reductase still required CM components.

In *S. oneidensis*, the menaquinone pool links primary dehydrogenases (i.e. formate or NADH dehydrogenase) to CymA [34], [35]. The fact that CymA was responsible for 85% of the total current flowing from electrodes into the cell for fumarate reduction combined with its known interaction with the menaquinone pool, suggested a possible role for the menaquinone pool in linking electron flux back out to another CymA protein, and finally to FccA (Fig. 4). A previously characterized mini Tn-10 insertion in the *menC* gene (o-succinylbenzoate synthase [29]), which is required for menaquinone biosynthesis [37], [38] was used in these studies. The specific current of *menC* mutant cells was only 5% (+/−2% n = 3) compared to wild type (Fig. 4). Heme stain profiles of whole cell extracts separated by SDS-PAGE showed that expression of CymA and other Mtr proteins was not affected by the *menC* insertion (data not shown). Taken together, it was evident that the majority of inward electron flux from the electrode to fumarate reductase required both OM and periplasmic components of the Mtr respiratory pathway, and passed into the menaquinone pool before re-entering the periplasm.

### Riboflavin enhances electrode-dependent inward electron flux to FccA but cannot replace lost periplasmic or cytoplasmic components

Previous experiments have shown that soluble redox shuttles (e.g. riboflavin, FAD, and FMN) significantly enhance turnover rates of cytochromes reducing insoluble iron oxides, even at the 0.5−1 µM levels that typically accumulate in *Shewanella* planktonic cultures [14], [39] and electrode-attached biofilms [8], [9], [40]. Similar to what has been observed with pure cytochromes [14] and whole cells [9], addition of riboflavin (1 µM) to wild type cells stimulated electron flux over 4-fold, with an average current density of 1.5+/−0.3 µA/µg (Fig. 4). However, addition of 1 µM riboflavin to Δ*mtrB* only raised the rate of fumarate-specific electron uptake to 0.13+/−0.05 µA/µg (Fig. 4). The inability of riboflavin to restore Δ*mtrB* underscored the importance of the outer membrane conduit in delivering electrons to the periplasm, and showed that soluble shuttling from the electrode into the periplasm could not replace this direct pathway, even at concentrations of 1 µM.

Another interaction that could have been enhanced by flavin shuttling was the slow rate of electron transfer attributed to MtrA-FccA remaining in Δ*cymA* and the *menC* mutant. Addition of 1 µM riboflavin to Δ*cymA* and the *menC* mutant had a larger stimulatory effect on the rate of electron uptake compared to Δ*mtrB*, but remained well below the current density observed in wild type cells with riboflavin (Fig. 4). Thus the electron uptake rate for Δ*cymA* and the *menC* mutant (∼20% of wild type) could represent an upper boundary for electron transfer between MtrA and FccA in the presence of physiological flavin levels. Addition of riboflavin to periplasmic cytochrome mutants (Δ*mtrA*, Δ*PEC*) had no stimulatory effect; further supporting the model that riboflavin shuttling from electrodes into the periplasm could not significantly contribute to the overall reaction.

**Figure 5:**
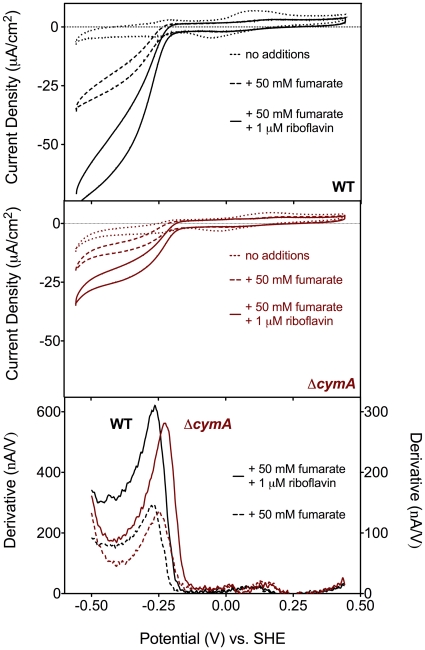
Riboflavin causes a shift in the potential required for reductive electron flow into Δ*cymA*. Representative cyclic voltammograms (1 mV/s) of A) *S. oneidensis* MR-1 with no additions (dotted line), 50 mM fumarate (dashed line), and 50 mM fumarate +1 µM riboflavin (solid line) for (A) wild type thin films and (B) Δ*cymA* thin films. (C) Derivative plots showing midpoint potentials for WT (black traces) and Δ*cymA* (red traces). The midpoint potential for the *menC* mutant was similar to Δ*cymA* (see text for details).

A final analysis aimed at elucidating the role of flavins in electron uptake was cyclic voltammetry. Addition of 1 µM riboflavin to wild type cells reducing fumarate increased the limiting current, but did not alter the onset potential or midpoint potential of the catalytic wave (Fig. 5). Thus electrochemical responses were consistent with an increased turnover of the overall pathway (e.g., electrode-to-outer membrane conduit), and the shape of the wave reflected no significant change in the driving force required to reduce FccA. Voltammetry of Δ*cymA* in the presence of flavins confirmed the weak stimulation observed in poised-potential experiments (Fig. 5), but also revealed a shift in the potential dependence of the catalytic reaction. The midpoint potential of the catalytic wave increased by +50 mV when 1 µM riboflavin was added to biofilms actively reducing fumarate; the *menC* mutant exhibited a similar shift (data not shown). The positive shift in potential further suggested that flavins enabled electron transfer to FccA, in the absence of CymA, via a pathway unique from what was active in wild type cells.

## Discussion

In this work, voltammetric techniques typically used to examine electron transfer from bacteria to surfaces [30], [40]–[42] were used to demonstrate the ability of electrode surfaces to drive reductive reactions in *S. oneidensis*. Comparison of multiple mutants provided evidence for the role of specific proteins from the Mtr respiratory pathway in this process.

The main pathway for outward electron flux to insoluble metals, electron shuttles, and electrodes has repeatedly been shown to involve the MtrCAB protein complex. In a recent study by Hartshorne et al. [4], MtrC and MtrA were shown to interact through MtrB. Interaction between MtrC and MtrA, and consequently facile electron transfer across the OM, has never been shown in the absence of MtrB, since MtrB is required for the proper localization of MtrA [4], MtrC and OmcA [31]. In our electron uptake assay Δ*mtrB* had ∼12% of the activity of wild type, showing the primary role of this conduit.

In addition to MtrCAB, the *S. oneidensis* genome encodes modular paralogs (i.e. MtrDEF) of the Mtr respiratory pathway [32]. As such, residual activity in Δ*mtrB* was either due to individual Mtr pathway paralogs, or to complete alternative complexes in the membrane. For example, MtrB paralogs such as MtrE can partially rescue Δ*mtrB* (Coursolle and Gralnick, unpublished data). An alternative hypothesis is that a complete conduit comprised of MtrDEF could be responsible for the remaining activity in Δ*mtrB*.

Deletion of periplasmic components provided evidence against the complete MtrDEF conduit hypothesis, as mutants lacking *mtrA* were capable of only ∼3% of wild type rates. This demonstrated that only 3% of electron uptake could be attributed to alternative complexes such as MtrDEF. The low residual effect of other periplasmic cytochromes *mtrD*, *cctA*, *dmsE*, and *SO4360* was also consistent with recent findings [32] where MtrD, CctA and DmsE were found to play a minor role in insoluble iron reduction in the absence of primary periplasmic electron carriers (i.e. MtrA). Furthermore, while a stable MtrAB subcomplex can form in the absence of MtrC, MtrB does not form a complex with MtrC in the absence of MtrA [4]. Thus, MtrA is required for rapid electron transfer into the periplasm, primarily as part of the MtrCAB complex, and other OM-spanning complexes do not play a major role under these conditions.

The requirement of CymA and the menaquinone pool for electrode-dependent fumarate reduction was determined through mutant analysis and was corroborated by thermodynamic evidence. In slow scan rate CV analysis, the catalytic waveshape from wild type films (Fig. 2) was similar to purified protein films of FccA [43], [44] with an onset potential and secondary boost well below the fumarate/succinate redox couple. In wild type films, however, the midpoint potential was centered at −270 mV *versus* SHE (Fig. 5), nearly 100 mV more negative than what is required to drive reduction by purified FccA. The fact that disruption of CymA and menaquinone biosynthesis shifted the onset and midpoint potential of fumarate reduction more positive (Fig. 5) indicated that electron flow through CymA and the menaquinone pool was partially responsible for this stronger driving force requirement.

Recently, Schuetz et al., [33] conducted *in vitro* experiments to examine putative pathways of electron transfer within the periplasmic space. Kinetic assays found that direct and reversible electron transfer could occur between purified MtrA and FccA [33]. However, rapid electron transfer between CymA and MtrA was also observed, and was determined to be 1.4-fold faster than the MtrA:FccA couple. Our study revealed a similar bias favoring the CymA:MtrA pathway and supports a model where the main conduit into the cell prefers MtrA reducing CymA, and that *in vivo* MtrA reduces FccA as a secondary reaction (<15% of activity), either by direct transfer or via periplasmic intermediates. For example, riboflavin was able to accelerate electron transfer in a Δ*cymA* strain (Fig. 4). Further evidence that flavins were altering the overall pathway was found in the shift in driving force needed to reduce fumarate when riboflavin was provided to the *cymA* mutant. The requirement for MtrA also confirmed that FccA was unable to be reduced by riboflavin shuttling from the electrode and across outer membrane at appreciable rates.

While the Mtr pathway in *S. oneidensis* is required for optimal electron transfer both into and out of the cell, this is in contrast to recent data suggesting *Geobacter sulfurreducens* exploits two separate pathways. Through gene deletion and gene expression studies, Strycharz et al. [45] showed that electron transfer into *Geobacter* biofilms was independent of major outer membrane cytochromes (OmcZ, OmcB, OmcST, and OmcE) required for electron transfer out of the cell, and was instead dependent upon a putative monoheme *c*-type cytochrome [45]. Therefore, differences in electron transfer pathway complexity between *Shewanella* and *Geobacter* likely reflect their very different environmental niches and electron transfer strategies.

### Conclusions

**Figure 6:**
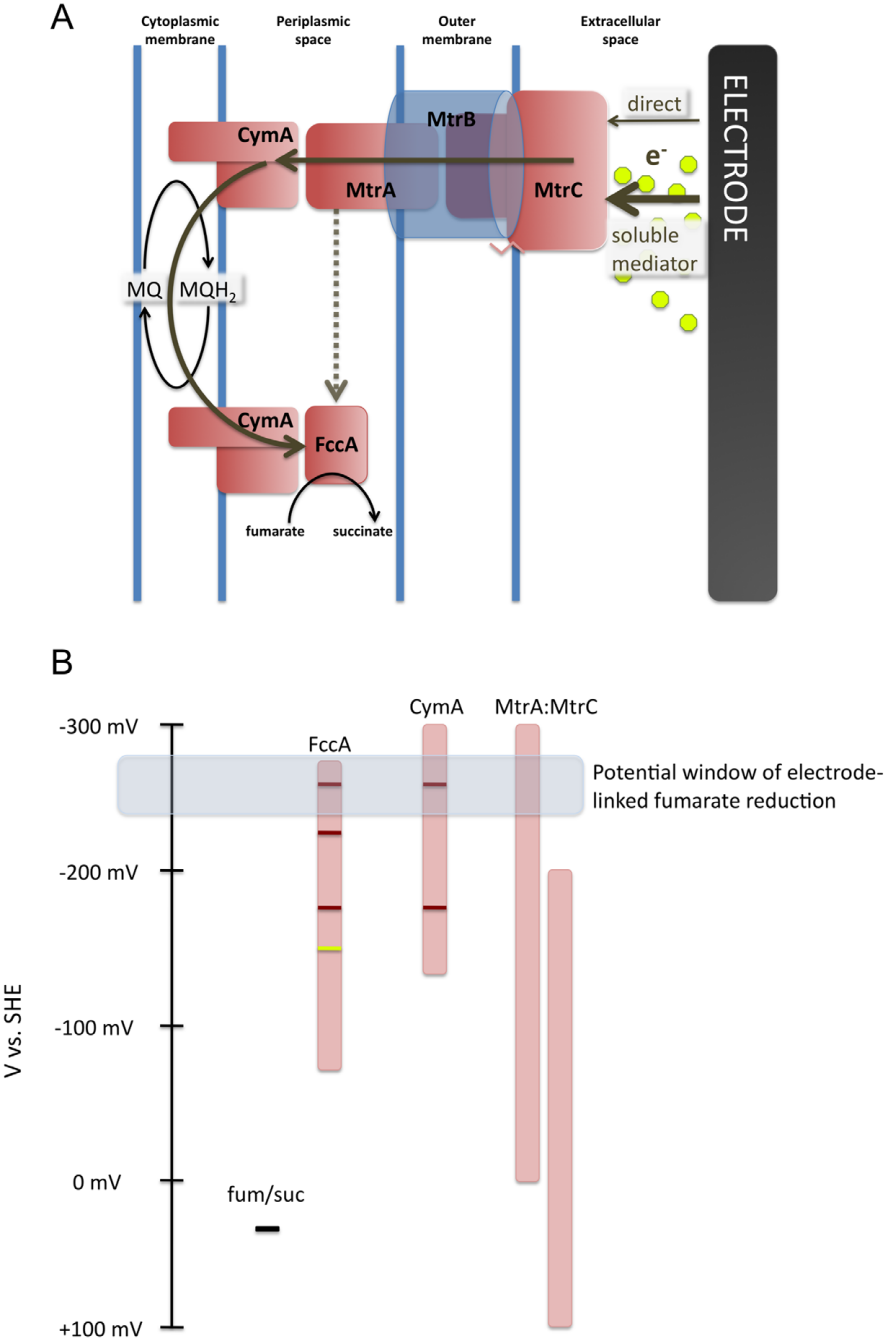
A model for reversible electron transfer through the Mtr respiratory pathway in *S. oneidensis* MR-1. (A) Electrons generated at the electrode surface are transferred to MtrC. MtrC then transfers electrons to MtrA by interacting through MtrB. From MtrA electrons are passed to CymA and through the menaquinone pool to a second CymA interacting with FccA. Approximately 85% of inward electron flux is dependent on flow through the menaquinone pool, while 15% relies on transfer from MtrA to FccA. Multi-heme cytochromes are in red and non-heme proteins in blue. (B) Redox potential windows for components involved in electrode-dependent fumarate reduction in *S. oneidensis*. Dark red lines represent midpoint potentials for specific hemes within CymA or FccA. The yellow line represents the midpoint potential of the FAD cofactor of FccA (data compiled from [4], [11], [16], [26], [43], [44], [46]).

Here we report genetic, electrochemical, and thermodynamic evidence for the reversibility of the Mtr electron transfer pathway in *S. oneidensis* with a surprising requirement for CymA and the menaquinone pool in electrode-dependent reduction of fumarate by whole cells. Examination of reverse electron flow driven by an electrode also afforded mechanistic insights to periplasmic electron transfer in *S. oneidensis*, supporting distinct respiratory units comprised of CymA:MtrA and CymA:FccA *in vivo* (Fig. 6). The ability to utilize electrodes for extracellular reduction of the quinone pool suggests the potential to catalyze net reductions within the cell, which would also consume intracellular protons, and generate a proton motive force. The fact that the Mtr conduit alone can create this linkage, combined with recent functional expression of MtrCAB in *E. coli* [17] suggests a role for this pathway in engineered microbial electrosynthesis [18].
